# Linking Gene Expression in the Intestine to Production of Gametes Through the Phosphate Transporter PITR-1 in *Caenorhabditis elegans*

**DOI:** 10.1534/genetics.116.188532

**Published:** 2016-07-21

**Authors:** Zita Balklava, Navin D. Rathnakumar, Shilpa Vashist, Peter J. Schweinsberg, Barth D. Grant

**Affiliations:** * School of Life and Health Sciences, Aston University, Birmingham, B4 7ET, United Kingdom; † Department of Molecular Biology and Biochemistry, Rutgers University, Piscataway, New Jersey 08854

**Keywords:** *Caenorhabditis elegans*, sodium-dependent phosphate transporter, PiT, germline signaling, yolk, phosphate sensing

## Abstract

Inorganic phosphate is an essential mineral for both prokaryotic and eukaryotic cell metabolism and structure. Its uptake into the cell is mediated by membrane-bound transporters and coupled to Na^+^ transport. Mammalian sodium-dependent Pi cotransporters have been grouped into three families NaPi-I, NaPi-II, and NaPi-III. Despite being discovered more than two decades ago, very little is known about requirements for NaPi-III transporters *in vivo*, in the context of intact animal models. Here we find that impaired function of the *Caenorhabditis elegans* NaPi-III transporter, *pitr-1*, results in decreased brood size and dramatically increased expression of vitellogenin by the worm intestine. Unexpectedly, we found that the effects of *pitr-1* mutation on vitellogenin expression in the intestine could only be rescued by expression of *pitr-1* in the germline, and not by expression of *pitr-1* in the intestine itself. Our results indicate the existence of a signal from the germline that regulates gene expression in the intestine, perhaps linking nutrient export from the intestine to production of gametes by the germline.

INORGANIC phosphate (Pi) is the second most abundant mineral in the human body, essential for prokaryotic and eukaryotic cell metabolism and structure. Due to the negative electrochemical potential across the cell membrane, Pi cannot cross the cell membrane by simple diffusion. The uptake of Pi into the cell in mammals is coupled to Na^+^ transport (Werner *et al.* 1994). Mammalian Na^+^-Pi cotransporters have been grouped into three families NaPi-I, NaPi-II, and NaPi-III, based on their sequence and structural similarity. The physiological role of NaPi-I remains to be fully established. NaPi-II plays a major role in renal Pi reabsorbtion (Werner *et al.* 1998). NaPi-III transporters were originally identified as retroviral receptors for rat amphotropic virus and gibbon ape leukemia virus (Miller *et al.* 1994; Miller and Miller 1994).

NaPi-III (also called the Pit family) family proteins have homologs in various organisms from bacteria to humans. The *Caenorhabditis elegans* genome encodes six predicted NaPi-III genes, also named phosphate permeases (Werner and Kinne 2001). None of these have been characterized on a functional level. Type III phosphate transporters share comparable membrane topologies, including 8–12 transmembrane spanning regions.

Mouse knockout of PiT-1 was reported to be embryonic lethal (Festing *et al.* 2009; Beck *et al.* 2010). There is currently no information on PiT-2 knockout phenotypes. In budding yeast, the high-affinity Pi transport system has been shown to function under Pi-starved conditions and is composed of independently regulated Pho84p and Pho89p (Pattison-Granberg and Persson 2000). Additionally Pho89p has been demonstrated to be mainly active under alkaline pH (Zvyagilskaya *et al.* 2008).

At the messenger RNA (mRNA) level, members of NaPi-III transporter family are ubiquitously expressed in tissues, and their expression levels respond to extracellular Pi concentration (Werner *et al.* 1998). Although discovered two decades ago, to date there is very limited data available regarding the tissue and subcellular distribution of NaPi-III transporters at the protein level. Importantly, recent evidence demonstrates an important role of NaPi-III transporters in bone Pi metabolism and vascular calcification (Lau *et al.* 2010).

We have previously described an *in vivo* assay for the trafficking of fluorescently tagged yolk protein YP170a, encoded by the *vitellogenin-2* (*vit-2*) gene (Grant and Hirsh 1999). Like endogenous YP170, the YP170::GFP fusion protein is synthesized in the *C. elegans* intestine and secreted basolaterally into the body cavity. From the body cavity, it is efficiently endocytosed by the oocytes using the oocyte-specific RME-2 yolk receptor (Grant and Hirsh 1999). Here, we report on a mutant isolated in our forward genetic screen for abnormally high levels of YP170::GFP accumulation in the body cavity, a phenotype usually associated with poor endocytosis of yolk by the oocytes. However, the unusual mutant described here does not affect yolk endocytosis, but rather increases expression of yolk protein genes in the intestine. Molecular cloning showed that this mutation impairs the function of the *C. elegans* NaPi-III transporter gene *pitr-1*. Surprisingly, only expression of *pitr-1* in the germline, but not the intestine, restores intestinal yolk protein gene expression to normal levels, implying the existence of a feedback mechanism linking gamete production in the germline to gene expression in the intestine needed to promote embryo production.

## Materials and Methods

### General methods and strains

Maintenance and genetic crosses of *C. elegans* strains were performed according to standard protocols (Brenner 1974). All strains of *C. elegans* were derived from wild-type (WT) Bristol strain N2. All strains were grown at 20°, unless otherwise stated. The following *C. elegans* strains were obtained from the *Caenorhabditis* Genetics Center: *unc-24*(e448)IV, *unc-44*(e362)IV, *C48A7.2*(ok2116) IV/nT1[qIs51](IV;V), *ppw-1(pk2505)I*, and *ppw-1(pk1425)*.

Transgenic strains *bIs1*(*vit-2*::*gfp*), *pwIs23*(*vit-2*::*gfp*), *pwIs88*(*vit-2*::*rfp*), and *pwIs116*(*rme-2*::*gfp*) have been described previously (Grant and Hirsh 1999; Balklava *et al.* 2007; Sato *et al.* 2008). *pwIs676* (p*pie-1*::*pitr-1*::*gfp*), *astIs1* (*pvha-6*::*pitr-1*::*gfp*), and *pitr-1(pw8[pitr-1*::*gfp])* are new strains from this work.

RNA-mediated interference (RNAi) feeding constructs were either obtained from the Ahringer library (Kamath and Ahringer 2003) or prepared from EST clones kindly provided by Yuji Kohara (National Institute of Genetics, Shizuoka, Japan) and cloned into the RNAi vector L4440 (Timmons and Fire 1998). RNAi was performed by the feeding method (Kamath and Ahringer 2003).

### Genetic mapping and molecular cloning

*b1028* was isolated in a screen described previously (Grant and Hirsh 1999). The *b1028* mutation was mapped between *unc-24* and *unc-44* of LG IV by classical three-point mapping. The *b1028* mutation was further narrowed down to reside between *F55G1* and *T12B3.3* by two-point mapping and snip-SNP analysis. To identify a candidate for *b1028*, we screened the predicted genes in the *C. elegans* genomic sequence in this region by RNAi for the YP170::GFP phenotype, and found that RNAi for the gene *C48A7.2* produced this phenotype in F_1_ generation. *C48A7.2* sequence was amplified from the *b1028* mutant by PCR and sequenced. Sequencing revealed a single nucleotide change at the splice site at the end of exon 7 (G2056A).

The *b1028* mutation was originally isolated in a mutagenesis screen designed to discover novel receptor-mediated endocytosis genes and was therefore assigned to the gene name *rme-10* (WormBase, WS253). Our subsequent analysis indicated that *b1028* does not cause impaired yolk endocytosis, but rather increases yolk production. Therefore we renamed the gene *pitr-1* to reflect its identity as a sodium-dependent phosphate transporter.

### Plasmids and transgenic strains

All cloning was performed using the Gateway cloning system (Invitrogen, Carlsbad, CA). All the destination vectors were adapted for the Gateway cloning system by insertion of appropriate Gateway cassettes.

To express the *pitr-1p*::*gfp* fusion, a 5.2-kb upstream sequence of *pitr-1* was amplified and cloned into pDONR221 and then transferred into pPD95.75-Gtwy by Gateway recombination. To express functional GFP-tagged PITR-1, *pitr-1* coding sequences from the start to the stop were amplified from genomic DNA and cloned into pDONR221 by Gateway cloning. GFP(S65C) with *C. elegans* introns was then PCR amplified from plasmid pPD117.01 (gift of A. Fire) and cloned into a *Spe*I site introduced by site-directed mutagenesis and transferred into vector pID2.01B (*pie-1* promoter) for germline expression (gift from Geraldine Seydoux) (Gallo *et al.* 2008) or vector pPS2 (*vha-6* promoter) for intestine-specific expression, using the Gateway LR reaction. This inserted GFP after Q522, within the predicted large extra cytosolic loop of *pitr-1* (between transmembrane domains TM7 and TM8). All integrated transgenic lines were obtained by the microparticle bombardment method (Praitis *et al.* 2001). Destination vectors containing *Cbr-unc-119* gene from *C. briggsae* were bombarded individually, but destination vectors without integrated *unc-119* gene were cobombarded with plasmid MM016B encoding the WT *unc-119* gene into *unc-119(ed3)* mutant worms.

CRISPR-Cas9-mediated gene editing was performed as described in Paix *et al.* (2015) using the co-CRISPR method. The guide CRISPR targeting RNA (crRNA) (auaguugguugaucgagaag) and primers for amplifying the GFP repair template (gaaaatatcttgaatccgacaataacggtcaacctATGAGTAAAGGAGAAGAACTTT and catgtactgactacaatagttggttgatcgagaagTTTGTATAGTTCGTCCATGC) were synthesized by IDT (Coralville, IA). Homology arms added to GFP are underlined. GFP (S65T) with *C. elegans* introns was amplified from plasmid pPD114.108 (gift of A. Fire).

### RNAi

RNAi was performed as described by Timmons and Fire (1998) with minor modifications. Synchronized L1 animals were added to RNAi plates and incubated for 72 hr until they reached adulthood. Adult worms were then transferred to fresh RNAi plates, allowed to lay eggs for 6 hr, after which they were removed, and the resulting eggs left to grow until they reached adulthood. Both P_0_ and F_1_ adults were used for SDS/PAGE and WB to analyze YP170 expression.

### Immunoblotting and antibodies

For immunoblotting, lysates were prepared from 20 hand-picked adults (24 hr after L4) by boiling animals in 25 μl of Laemmli sampling buffer and subjected to SDS/PAGE and Western blotting. Membranes were blocked with 3% (w/v) BSA in PBS and probed with either rat anti-YP170 (1:10,000 dilution) (kindly provided by T. Blumenthal (University of Colorado) or monoclonal mouse anti-actin JLA20 (1:1,000 dilution) (Developmental Studies Hybridoma Bank, Iowa City, IA) primary antibodies diluted in 3% (w/v) BSA in PBS/0.5% Tween 20 at 4° overnight. Following washing, the membranes in PBS/0.5% (v/v) Tween 20 the membranes were incubated for 2 hr at room temperature with HRP-conjugated secondary antibodies (1:2000 dilution) (Sigma, St. Louis, MO). The signal was detected using Pierce ECL 2 Western blotting substrate (Thermo Scientific, Waltham, MA).

### Microscopy

To observe transgenes in live worms, worms were mounted on 2% (w/v) agarose pads with 100 mM tetramisol (Sigma) in M9 buffer. To quantify yolk level in embryos, worms were first dissected, mounted on agarose pads in M9 buffer, and imaged. Fluorescence images were obtained using either an Axiovert 200 M (Carl Zeiss MicroImaging, Munich, Germany) microscope equipped with a digital CCD camera (C4742-95-12ER, Hamamatsu Photonics, Hamamatsu, Japan) and Metamorph software (Universal Imaging, Downingtown, PA) and then deconvolved with AutoDeblur software (AutoQuant Imaging) or DMI4000B (Leica Microsystems, Wetzlar, Germany) microscope equipped with CCD Leica DFC360 FX camera using Leica application suite AF software.

### Isolation of RNA and complementary DNA synthesis

Total RNA was isolated from 6-cm plates containing synchronized young adult hermaphrodites using TRI reagent (Sigma) according to the manufacturer’s instructions. All samples were DNase I treated (Fermentas). First-strand complementary DNA (cDNA) was synthesized from 2 μg RNA using an oligo(dT) primer and a Moloney murine leukemia virus reverse transcriptase (Fermentas) for 1 hr at 42°.

### Real-time RT-PCR

The primers were designed and purchased from PrimerDesign (Southampton, UK). Semiquantitative RT-PCR was carried out using a Stratagene Mx3000P real-time cycler (Stratagene, La Jolla, CA) using the SYBR Green Precision Master Mix (PrimerDesign). Each reaction contained: 10 μl of the Precision Master Mix, 1 μl of forward and reverse primer mix, and 5 μl cDNA (1:10 RNA dilution), to a final volume of 20 μl. All reactions were set up in triplicate for each primer set. The cycling conditions were as follows: enzyme activation 95° for 10 min, followed by 40 cycles of 15 sec at 95°, and 60 sec at 60°. Following the final cycle, melting curve analysis was performed to examine the specificity in each reaction tube (absence of primer dimers and other nonspecific products) for 10 sec from 55° to 95°. A single melt peak for each reaction confirmed the identity of each PCR product. Each assay included a no-template control and RNA control for every primer pair. The threshold cycle (Ct) values were used to analyze the quantitative PCR (qPCR) data. Comparisons were made between the average crossing point (Ct) obtained from the *vit-2* gene and the housekeeping gene *eif-3.C* (ΔCt). ΔCt values were calculated for each sample. The level of each transcript relative to housekeeping gene levels could then be calculated from the equation 2-ΔΔCt where ΔΔCt is the ΔCt value of the gene of interest transcript normalized to reference transcript (Livak and Schmittgen 2001).

### Data availability

Strains are available upon request. The authors state that all data necessary for confirming the conclusions presented in the article are represented fully within the article.

## Results

### A *C. elegans* mutant with abnormal yolk expression

**Figure 1:**
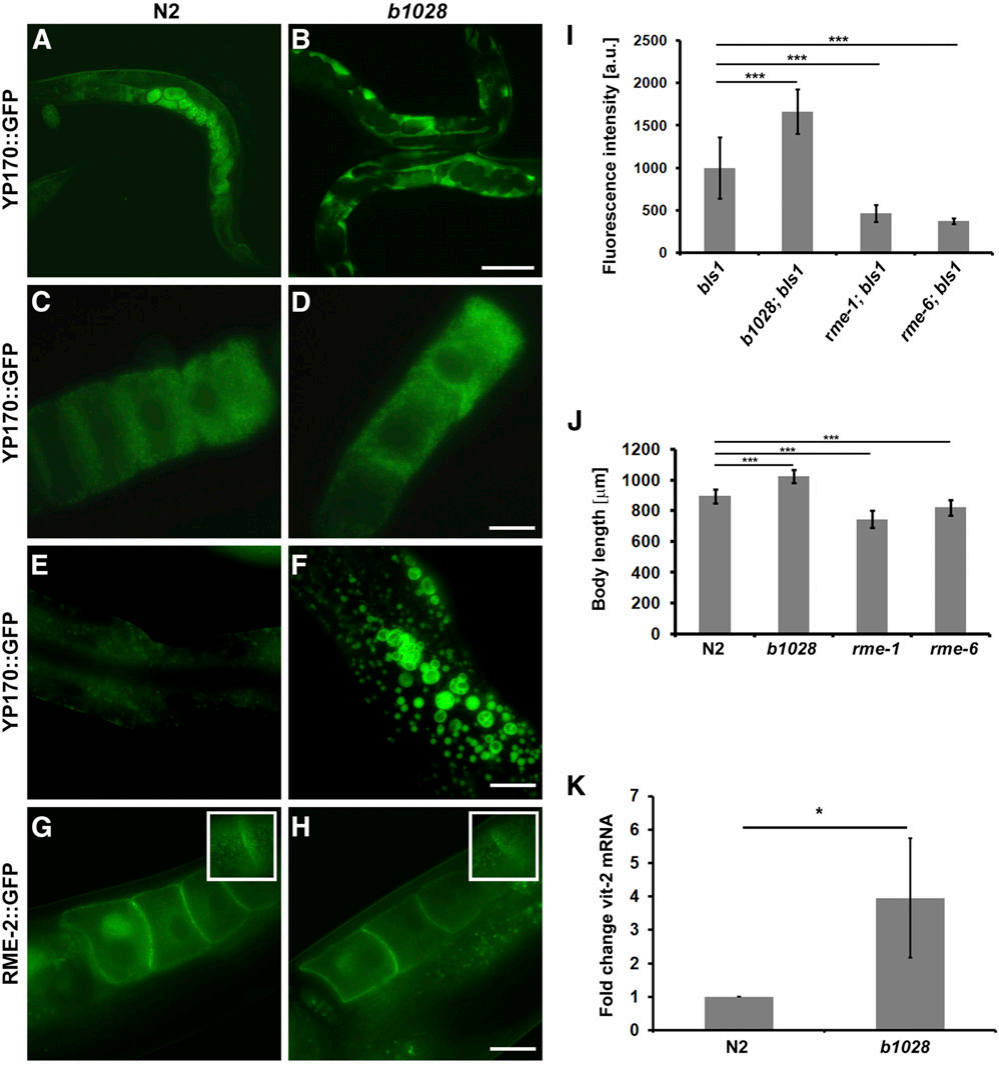
Overaccumulation of yolk in *b1028* mutants. (A–F) YP170::GFP trafficking in adult hermaphrodites. In WT worms, YP170::GFP is efficiently endocytosed by oocytes (A). In the *b1028* mutant worms, YP170::GFP accumulation in the body cavity is greatly increased (B). (C and D) Both WT and *b1028* mutant worms show comparable levels of YP170::GFP in oocytes. WT worms show detectable levels of YP170::GFP in the intestine, while *b1028* worms show increased accumulation of enlarged YP170::GFP positive vesicles in the intestine (E–F). (G and H) The localization of yolk receptor RME-2::GFP is unaltered in *b1028* mutant oocytes. Images were taken through the middle focal plane of oocytes. Insets show enlarged top focal plane images of oocytes, demonstrating RME-2::GFP positive cortical endosomes. Bars (A and B), 100 μm; (C–F), 20 μm. (I) YP170::GFP fluorescence quantification in one- and two-cell embryos, results are expressed as arbitrary units of fluorescence intensity ± SD, asterisks indicate statistical differences (*t*-test, *P* ≤ 0.001). (J) Size measurement of WT and various *rme* mutant worms (*n* ≥ 12), results are expressed in micrometers ± SD, asterisks indicate statistical differences (*t*-test, *P* ≤ 0.001). (K) Real-time PCR analysis of *vit-2* expression in WT and *b1028* mutant worms. *eif-3.C* was used as a reference mRNA. Results are expressed as a fold change ± SD; asterisks indicate statistical differences (*t*-test, *P* ≤ 0.05).

Allele *b1028* was isolated in a previously described genetic screen designed to discover mutants that were defective in receptor-mediated endocytosis (RME) of YP170::GFP in oocytes (Grant and Hirsh 1999). In the *b1028* mutant YP170::GFP accumulated in the body cavity, a phenotype that typically indicates reduced yolk uptake by oocytes (Figure 1, A and B). Careful analysis of the yolk phenotype of *b1028* revealed that, although mutant worms had increased accumulation of YP170::GFP in the body cavity, the oocytes and embryos also contained high levels of YP170::GFP (Figure 1, A–D), suggesting that receptor-mediated endocytosis was not impaired in this mutant. We also observed an increased accumulation of enlarged yolk containing vesicles in the intestine of *b1028* mutant worms (Figure 1, E and F) that could result from augmented yolk synthesis.

To analyze defects in YP170::GFP distribution in greater detail, we measured the fluorescence intensity of endocytosed YP170::GFP in one- and two-cell embryos and compared it with WT and well-characterized *rme* mutant worms. As expected, the amount of YP170::GFP was significantly reduced in *rme-1* and *rme-6* mutant embryos when compared to the WT background. In contrast, *b1028* mutant worms had significantly elevated YP170::GFP levels in embryos when compared to the WT (Figure 1, A–D, see also Figure 5I for total yolk levels). Importantly, the distribution of GFP-tagged yolk receptor RME-2::GFP in *b1028* worms appeared to be normal. RME-2::GFP localization to the plasma membrane and cortical endosomes of the oocytes was unaffected, further indicating normal endocytic trafficking in the oocytes of *b1028* mutants (Figure 1, G and H and insets). YP170 expression was not detected in *b1028* male worms, indicating that the effect is specific to hermaphrodites (Supplemental Material, Figure S2). In addition, unlike *rme-1* and *rme-6* mutant worms, which are shorter than WT animals, *b1028* worms displayed longer than WT bodies (Figure 1J). The brood size of *b1028* mutant worms at 20° was also significantly reduced (140 ± 23, *n* = 20) compared to the WT worms (263 ± 31, *n* = 20) (Figure 5H). We found that at least part of the decrease in brood size may be due to defective spermatogenesis, as crossing *b1028* hermaphrodites with N2 males rescued the low brood size of *b1028* animals (261 ± 57, *n* = 16). Together these results suggested that changes in YP170::GFP distribution in *b1028* mutant worms might result from increased yolk protein synthesis and clearly did not reflect impaired yolk endocytosis.

Next, we sought to investigate whether the yolk phenotype in *b1028* worms is a result of elevated yolk expression. We performed a semiquantitative RT-PCR analysis where we analyzed endogenous *vit-2* mRNA levels, comparing WT and *b1028* mutant animals, normalized to the *eif-3.C* reference mRNA. Real-time RT-PCR results revealed that there is a significant increase (*P* < 0.05) in *vit-2* mRNA levels in *b1028* mutants compared to WT, indicating that endogenous *vit-2* gene expression is altered (Figure 1K).

### b1028 mutates sodium-dependent phosphate transporter PITR-1

**Figure 2:**
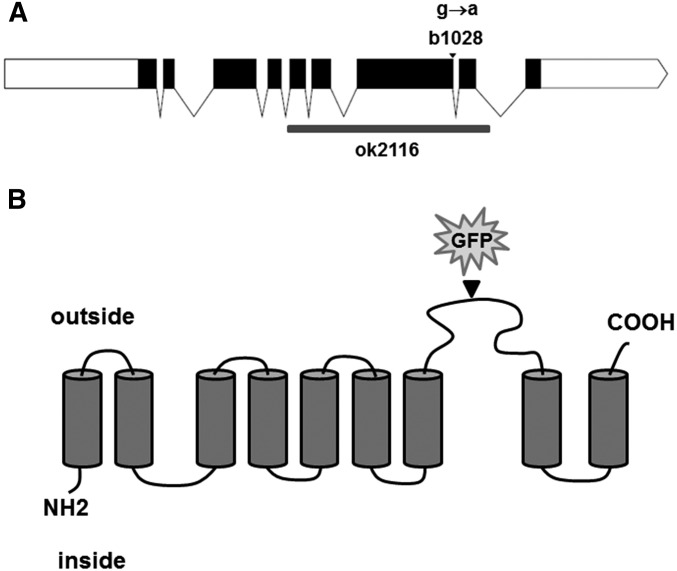
Predicted membrane topology of *C48A7.2*. (A) Diagram of the *C48A7.2* locus. Exons are in black; *b1028* mutation is indicated with an arrowhead; *ok2116* deletion is indicated with a gray box. (B) Putative topology of PITR-1 protein, based on TM domain prediction; insertion site of GFP into rescuing transgene is indicated by an arrowhead.

We mapped the *b1028* using standard methods to the genomic region between snip-SNPs *T12B3* and *F55G1* on LG IV. This region included 31 genes. To identify which of these genes is mutated in *b1028* animals, we first tested each for the increased yolk phenotype by RNAi, using transgenic worms expressing YP170::GFP in the RNAi sensitized mutant background *rrf-3(pk1426)* (Balklava *et al.* 2007). One of the RNAi knockdowns tested, targeting gene *C48A7.2*, produced an increase in YP170::GFP level after RNAi. *C48A7.2* was sequenced in the *b1028* mutant, and a G-to-A missense mutation (G2056A) was found at the splice site at the end of exon 7 in *C48A7.2* (Figure 2A).

**Figure 3:**
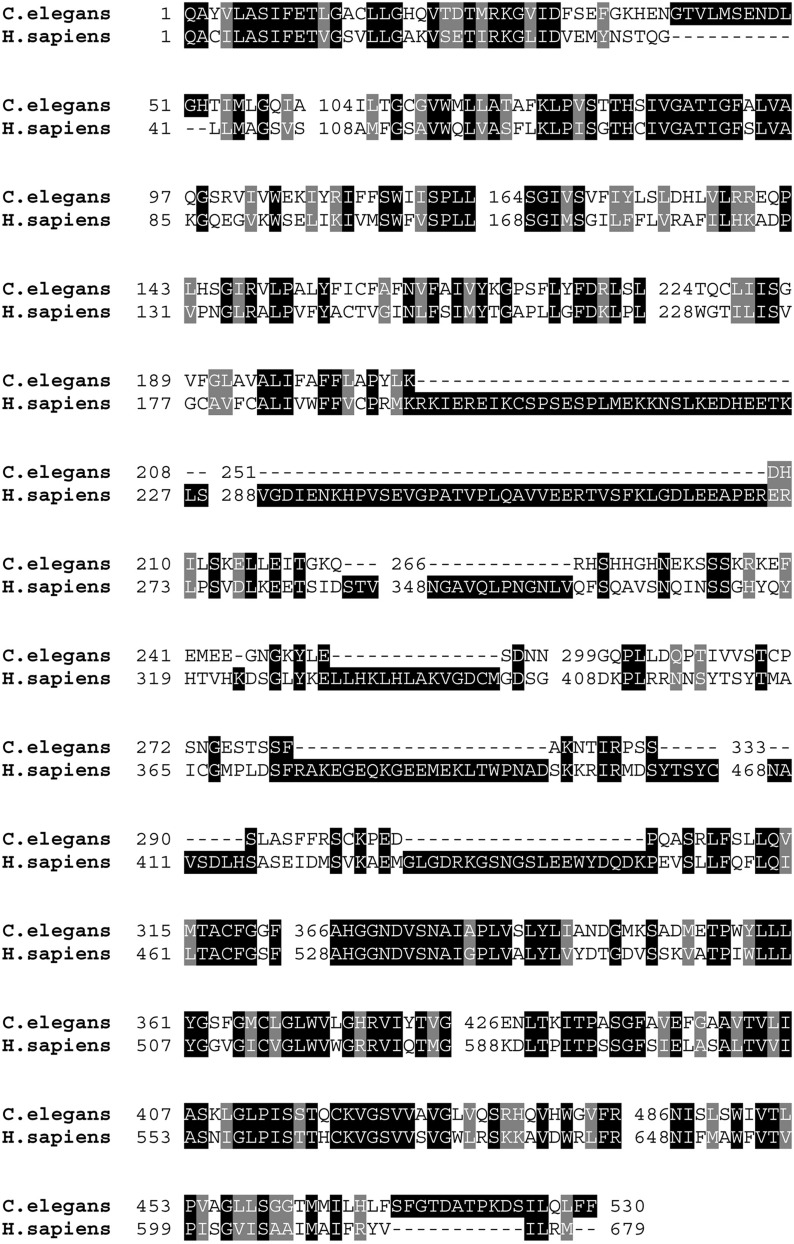
Sequence similarity of worm and human type III sodium-dependent phosphate transporters. Comparison of *C48A7.2* with ortholog from human (GenBank NP 005406) reveals 42% identity.

*C48A7.2* gene encodes a sodium-dependent phosphate transporter most closely related to human class III phosphate transporters [solute carrier family 20 (phosphate transporter), member 1; also known as SLC20A1, PIT1, GLVR1, PiT-1, and Glvr-1]. Therefore we named *C48A7.2pitr-1*, for phosphate transporter related 1. Sequence comparisons revealed 42% identity with its human homolog. The N and C termini showed more sequence similarity, with more variable identity level in between (Figure 3).

PITR-1 is a predicted 530-aa protein with nine transmembrane-spanning regions (Figure 2B). The G2056-to-A missense mutation in *pitr-1* at the end of exon 7 is expected to cause premature termination of translation, resulting in the loss of the last two transmembrane domains.

To further confirm that impaired function of *pitr-1* leads to the observed increase in vitellogenin expression, we further analyzed *pitr-1* by RNAi. We observed that N2 worms fed on either of two nonoverlapping *pitr-1*-targeted RNAi clones produced increased endogenous YP170 as judged by Western blot (Figure 5J and Figure S3). A similar increase in endogenous YP170 was also observed in *pitr-1(ok2116)* null animals and in heterozygous animals carrying one copy of *pitr-1(ok2116)* and one copy of *pitr-1(b1028)* (Figure S2).

We also found that a likely null allele of *pitr-1*, *ok2116*, which deletes most of the gene, can be maintained only in balanced heterozygotes. To determine the stage at which *pitr-1(ok2116)* animals arrest, we allowed 10 heterozygote *ok2116*/+ hermaphrodites to lay eggs on seeded NGM plates for 4 hr and then analyzed their progeny. Of 130 eggs, 79 never hatched. Of the 51 hatched embryos, 43 were *ok2116/+* heterozygotes, and the remaining 8 were homozygous for *ok2116*. Six *ok2116* larvae were slow growing but eventually reached adulthood, but then produced only a few embryos, none of which hatched.

### pitr-1 is most strongly expressed in the *C. elegans* germline

To determine where *pitr-1* is expressed in *C. elegans*, we used CRISPR-Cas9 gene editing to integrate GFP into the endogenous *pitr-1* gene (Paix *et al.* 2015). Following careful analysis of the *pitr-1* sequence, and comparison with human homologs, we decided to insert GFP in the large extracellular loop between transmembrane domains TM 7 and TM 8 (Figure 2B). This GFP-tagged allele of *pitr-1* is viable (98.6% ± 1%, *n* = 602) and fertile (brood size 278 ± 69, *n* = 15), indicating that it is functional.

**Figure 4:**
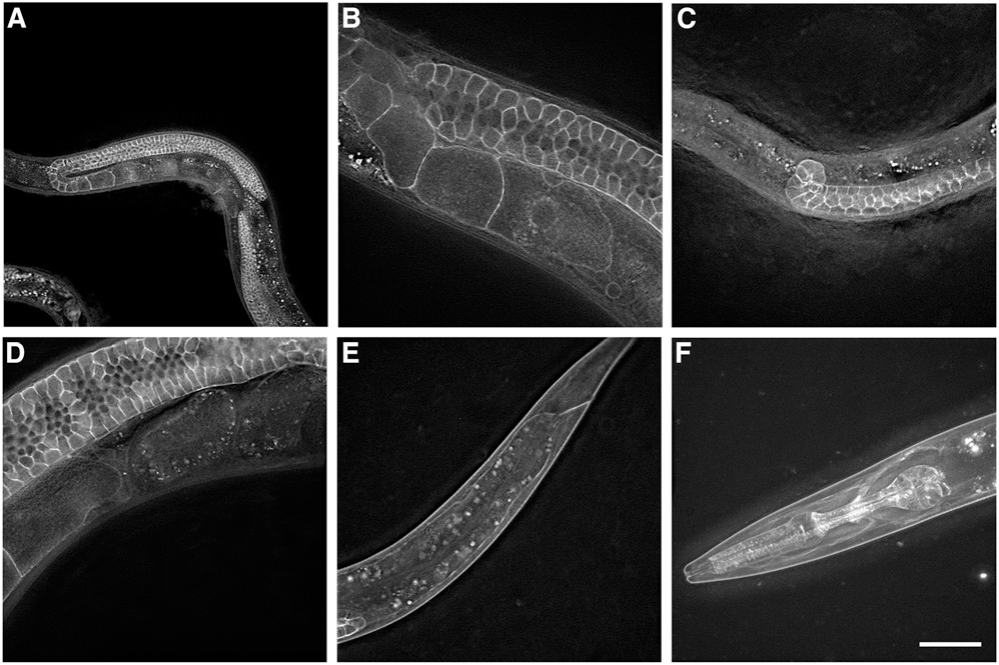
*pitr-1* is strongly expressed in *C. elegans* germline. Expression of endogenous PITR-1::GFP is indicated in (A and B) germline of young adults; (C) germline of larvae; (D) quickly degrading in embryos; (E) body-wall muscles of larvae; and (F) pharynx, head muscles, and muscle arms. Bar, 20 μm.

The strongest expression of PITR-1::GFP was in the germline, with clear labeling of the plasma membrane of all germ cells, from the distal tip to the oocyte closest to the spermatheca in adults, with apparent continuous labeling of developing germ cells at all larval stages (Figure 4). PITR-1::GFP on the surface of oocytes appeared to be internalized and degraded shortly after ovulation, similar to other oocyte-expressed transmembrane proteins such as RME-2. We also noted expression of PITR-1::GFP in the pharynx and head muscles, including muscle arms, of larvae and adults. Weak labeling of body wall muscles outside of the head was apparent in larvae but not adults. No clear labeling of the intestine by PITR-1::GFP was visible. These results are consistent with an important role for PITR-1 in germline development or function, but called into question whether *pitr-1* was expressed in the intestine where yolk gene expression takes place.

To determine if *pitr-1* might be expressed in additional tissues, perhaps at a lower level, we created transgenic animals expressing GFP driven by 5.2 kb of *pitr-1* upstream sequences (the predicted promoter region). We found that this construct expressed almost ubiquitously. GFP expression was observed in the pharynx, intestine, hypodermis, body-wall muscles, nerve ring, and many other neurons, oviduct sheath cells, and spermatheca (Figure S4). These results suggested that *pitr-1* may be expressed in the intestine at a low level, where it could directly influence yolk expression.

### pitr-1 is required in the germline to control YP170 expression in the soma

To more clearly define where PITR-1 functions with respect to yolk expression, we expressed a similar *pitr-1*::GFP fusion using tissue-specific promoters for the intestine or germline and assayed for rescue of *b1028* mutant yolk phenotypes. We generated transgenic worms expressing PITR-1::GFP driven by the *vha-6* promoter in the intestine and tested its rescuing properties in a *pitr-1(b1028)* mutant background expressing a YP170::RFP transgene. Although P*vha-6* driven expression of PITR-1::GFP was clearly visible in the intestine, the site of yolk synthesis, PITR-1::GFP expression in the intestine did not rescue the yolk phenotype of *pitr-1(b1028)* mutant worms (Figure S1).

**Figure 5:**
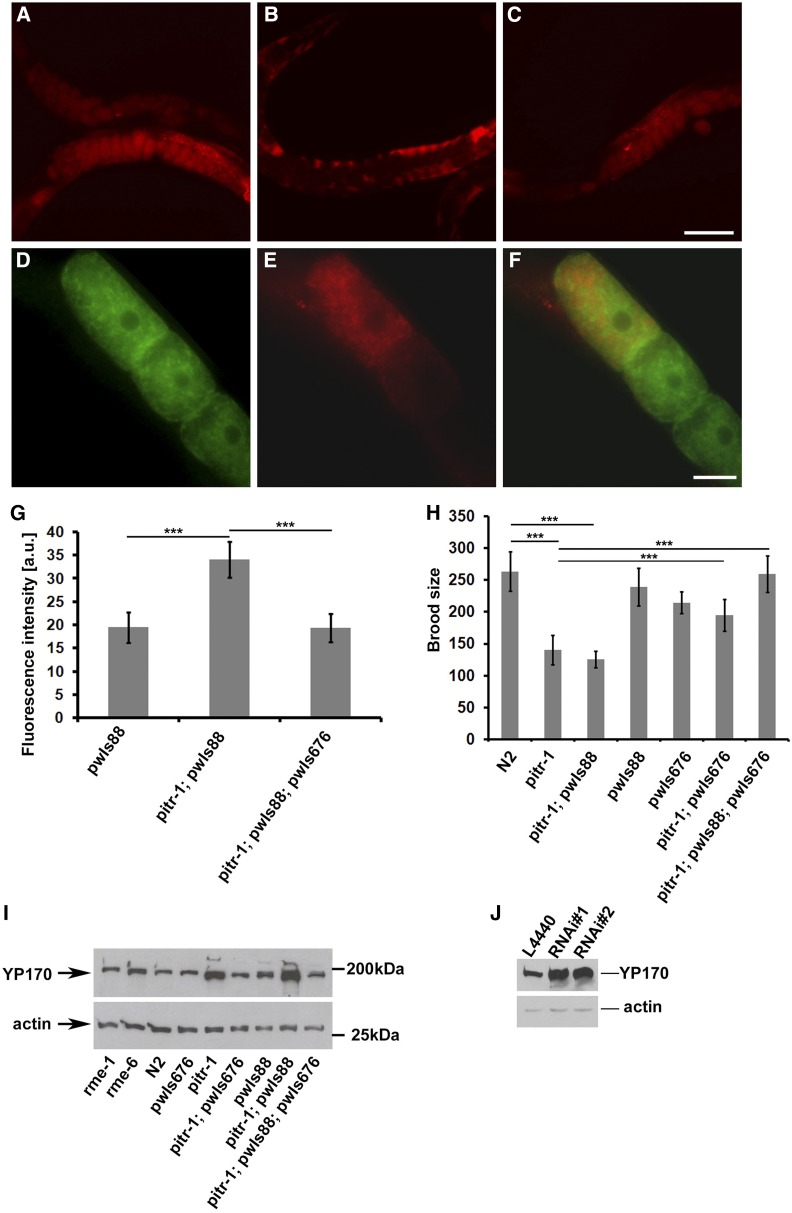
*pitr-1* rescue. (A–C) The defect in YP170 expression was rescued by expressing the *pie-1* promoter-driven PITR-1::GFP in the germline. (A) WT worms expressing pwIs88 (*vit-2*::*rfp*), (B) *pitr-1(b1028)* worms expressing *pwIs88*, (C) *pitr-1(b1028)* worms expressing *pwIs88* and rescuing transgene *pwIs676* (*pie-1p*::*pitr-1*::*gfp*). (D–F) Subcellular localization of PITR-1 and YP170 in *pitr-1(b1028)* rescued animal. Note that cell surface localization of PITR-1::GFP in these animals may have been quenched due to oxidation of the GFP(S65C) variant used in these experiments. (D) Localization pattern of PITR-1::GFP in oocytes, (E) localization of YP170::RFP in the oocytes, and (F) merged image showing that PITR-1::GFP and YP170::RFP positive structures do not colocalize. Bars (A–C), 100 μm; (D–F), 20 μm. (G) YP170::RFP fluorescence quantification in one- and two-cell embryos; results are expressed as arbitrary units of fluorescence intensity ± SD; asterisks indicate statistical difference (*t*-test, *P* ≤ 0.001). (H) Brood size count of WT, *pitr-1(b1028)*, and transgenic worms; for each bar *n* = 20 ± SD; asterisks indicate statistical difference (*t*-test, *P* ≤ 0.001). Decreased brood size of *pitr-1(b1028)* is rescued by expressing PITR-1::GFP in the germline. (I) SDS/PAGE and WB analysis of YP170 levels in WT and the indicated mutant genotypes. Increased accumulation of yolk in *pitr-1(b1028)* worms is rescued by expressing PITR-1::GFP in the germline. Actin was used as a loading control. (J) SDS/PAGE and WB analysis of YP170 levels in F_1_ N2 worms fed either with control (L4440) or two different RNAi clones (RNAi no. 1 and RNAi no. 2) targeting *pitr-1*. Actin was used as a loading control.

We next expressed the same *pitr-1*::GFP coding region using a well-characterized germline-specific promoter from the *pie-1* gene. Surprisingly, germline-specific expression of PITR-1::GFP rescued the *b1028* intestinal yolk expression defect, as judged by YP170::RFP fluorescence levels in the embryos and by Western blotting for endogenous YP170 and YP170::RFP in adult hermaphrodites (Figure 5, C and G). As expected, *rme-1* and *rme-6* mutant worms had YP170 levels similar to those of WT worms (Figure 5I). Expression of germline PITR-1::GFP in *pitr-1* mutant worms reduced both tagged and endogenous yolk expression to levels similar to WT (Figure 5I). Expression of germline PITR-1::GFP also rescued the reduced brood size of *pitr-1* mutant worms (Figure 5H).

We also sought to determine whether expression of PITR-1::GFP in the germline was sufficient to rescue the lethality/sterility of *pitr-1(ok2116)* deletion mutant worms. Expression of PITR-1::GFP in the germline was not sufficient to rescue *pitr-1(ok2116)* arrest/lethality, suggesting that the viability of *C. elegans* depends on *pitr-1* expression in more than one tissue.

Our rescue data suggest that *pitr-1* from the germline controls YP170 synthesis in the intestine. To test this idea further, we performed *pitr-1* RNAi in *ppw-1* mutant animals impaired for germline RNAi. In the *ppw-1* background, the *pitr-1* RNAi effect was mostly nullified (Figure S3). These results further support the hypothesis that *pitr-1* expression in the germline is required for proper regulation of YP170 expression in the intestine.

## Discussion

### pitr-1 mutants display increased yolk synthesis

The *pitr-1(b1028)* mutant was isolated in a genetic screen aimed to discover mutants that were defective in receptor-mediated endocytosis of YP170::GFP in oocytes (Grant and Hirsh 1999). Despite increased accumulation of YP170::GFP in the body cavity, careful analysis of *b1028* mutant worms revealed phenotypes not characteristic of an Rme phenotype. Although *b1028* worms displayed increased accumulation of YP170::GFP in the body cavity, they also displayed increased amounts of yolk uptake by oocytes as measured by YP170::GFP fluorescence intensity in one- and two-cell embryos (Figure 1I). qRT-PCR and Western blot analysis confirmed that in fact *pitr-1(b1028)* mutant worms synthesized more yolk protein than WT worms (Figure 1K and Figure 5I). The increased level of yolk production appeared to lead to increased uptake by oocytes, but likely exceeded their maximum capacity for uptake, leading to accumulation in the body cavity and intestine.

### C48A7.2 is a sodium-dependent phosphate transporter

*C48A7.2* codes for a sodium-dependent phosphate transporter most closely related to human class III phosphate transporters (Figure 3) and was therefore named *pitr-1*. Endogenous *pitr-1* tagged with GFP was strongly expressed in the germline and pharynx, with weaker signal observed in body-wall muscles, especially in the head (Figure 4). Analysis of the expression pattern of *pitr-1* promoter-driven GFP demonstrated ubiquitous tissue expression (Figure S4). PITR-1 homologs are found in a variety of organisms ranging from bacteria to mammals (Werner and Kinne 2001). They are ubiquitously expressed and their expression levels respond to extracellular Pi concentrations (Werner *et al.* 1998). Recent data demonstrate an important role of NaPi-III transporters in bone Pi metabolism and vascular calcification (Khoshniat *et al.* 2011 and references therein). Additionally, a PiT-1 knockout mouse has been demonstrated to be embryonic lethal (Festing *et al.* 2009; Beck *et al.* 2010). Our data in *C. elegans* are in agreement with the above findings, demonstrating *pitr-1* expression in multiple tissues and its essential role in development; however, strong expression of *pitr-1* in the germline highlights a key function in this tissue. The *b1028* allele contains a G-to-A missense mutation G2056A at the splice site at the end of exon 7 (Figure 2A) predicted to cause premature termination of translation prior to the last two transmembrane domains (Figure 2B). This is likely to impair phosphate transport by PITR-1, but *b1028* is probably a partial loss-of-function allele, since its other phenotypes are less severe than the *ok2116* deletion.

While our mutagenesis screen provided only one allele of *pitr-1*, *b1028*, we observed similar increased yolk synthesis phenotype with *pitr-1(ok2116)* null animals, as well as in animals where *pitr-1* was knocked down by RNAi (Figure S3 and Figure 5J), further supporting the relationship between *pitr-1* function and proper yolk expression. While our results indicate that *pitr-1* is required to regulate yolk synthesis, our rescue data suggested that *pitr-1* function in the germline is somehow linked to yolk synthesis in the intestine. RNAi data performed in germline RNAi defective mutant background further support our hypothesis that *pitr-1* plays a role in signaling between tissues (Figure S3). These results clearly indicate a nonautonomous role for *pitr-1* in controlling *vit-2* gene expression and suggests the existence of germline-to-intestine signaling.

Recently, a thorough study of *vit-2* promoter enhancer elements has revealed an unexpectedly complex network regulating *vit-2* transcription. Expression of *vit-2* in adult hermaphrodite intestinal cells is regulated through a concerted action of *elt-2* and *mab-3*. Additionally, the degree of *vit-2* expression is modulated by multiple signaling pathways, including insulin signaling and TGFβ/Sma/Mab pathways (Goszczynski *et al.* 2016). Our data suggest that *pitr-1* could similarly be involved in modulating *vit-2* transcription, possibly via nutritional status signaling from germline to intestine initiated by low Pi in germ cells.

The effect of high and low Pi in the diet has been shown to alter the growth of various organ systems (Khoshniat *et al.* 2011 and references therein). The phosphate transporters that may be involved in these processes are not clearly identified, but the ability of PiT to act as extracellular Pi “sensors,” and to respond to changes in extracellular Pi concentrations, positions them as potential mediators of Pi-induced signaling.

The importance of signaling between the germline and intestine is also supported by work showing that removal of germline stem cells results in increased fat accumulation in the intestine that is derived from unconsumed yolk (O’Rourke *et al.* 2009; Steinbaugh *et al.* 2015). Endocrine hormone signaling from germline to intestine in *C. elegans* has been previously reported to control life span (Spanier *et al.* 2010). Furthermore, loss of germline stem cells leads to overall transcriptional reprogramming in somatic tissues (Steinbaugh *et al.* 2015).

We deem it plausible that oocytes have sensors to measure whether they have acquired sufficient nutrient levels and a corresponding signaling pathway that feeds such information back to the intestine. Given the strong expression of PITR-1 on the surface of germ cells, PITR-1-mediated uptake likely represents a major source of phosphate for growing germ cells and embryos. If this source of phosphate is reduced, the production of alternate sources may be upregulated. Yolk may serve as an important source of phosphate for the germline and embryo.

Chicken and frog yolk phosvitin is highly phosphorylated and has been proposed to be a major source of phosphate in eggs and contributes to bone development in embryos (Mecham and Olcott 1948; Bergink and Wallace 1974; Li *et al.* 2014). Insect yolk is also highly phosphorylated (Don-Wheeler and Engelmann 1997). VIT-2 is a 1613-aa protein containing 117 Ser and 94 Thr, making the protein about 13% S/T. This may indicate that, as in other organisms, *C. elegans* phosphorylates yolk and uses it for phosphate transport. The high negative charge of phosphorylated phosvitin also allows it to carry metals such as calcium, magnesium, and iron (Jiang and Mine 2001; Samaraweera *et al.* 2011). Thus phosphorylated yolk transport may also provide essential metals for the embryo.

Alternatively, loss of *pitr-1* function could result in low ATP levels, which is interpreted as a general starvation signal by oocytes, resulting in signaling to the intestine to generally increase the transport of nutrients to the germline.

Low phosphate levels could also directly interfere with signaling pathways involving kinase cascades. For instance, in mammalian bone, increased Pi levels have been shown to selectively activate ERK1/2 signaling (Julien *et al.* 2009). Together these reports highlight the significance of signaling between tissues to support overall organismal physiology. Our data suggest that PITR-1 could play an important role in one or more of these signaling pathways.

## 
